# Simulations predict differing phase responses to excitation vs. inhibition in theta-resonant pyramidal neurons

**DOI:** 10.1152/jn.00160.2023

**Published:** 2023-08-23

**Authors:** Craig Kelley, Srdjan D. Antic, Nicholas T. Carnevale, John L. Kubie, William W. Lytton

**Affiliations:** ^1^Program in Biomedical Engineering, SUNY Downstate Health Sciences University and NYU Tandon School of Engineering, Brooklyn, New York, United States; ^2^Institute of Systems Genomics, Neuroscience Department, University of Connecticut Health, Farmington, Connecticut, United States; ^3^Department of Neuroscience, Yale University, New Haven, Connecticut, United States; ^4^The Robert F. Furchgott Center for Neural and Behavioral Science, SUNY Downstate Health Sciences University, Brooklyn, New York, United States; ^5^Department of Cell Biology, SUNY Downstate Health Sciences University, Brooklyn, New York, United States; ^6^Department of Physiology and Pharmacology, SUNY Downstate Health Sciences University, Brooklyn, New York, United States; ^7^Department of Neurology, SUNY Downstate Health Sciences University, Brooklyn, New York, United States; ^8^Department of Neurology, Kings County Hospital Center, Brooklyn, New York, United States; ^9^Aligning Science Across Parkinson’s (ASAP) Collaborative Research Network, Chevy Chase, Maryland, United States

**Keywords:** impedance phase, phase precession, phase roll, pyramidal neuron, resonance

## Abstract

Rhythmic activity is ubiquitous in neural systems, with theta-resonant pyramidal neurons integrating rhythmic inputs in many cortical structures. Impedance analysis has been widely used to examine frequency-dependent responses of neuronal membranes to rhythmic inputs, but it assumes that the neuronal membrane is a linear system, requiring the use of small signals to stay in a near-linear regime. However, postsynaptic potentials are often large and trigger nonlinear mechanisms (voltage-gated ion channels). The goals of this work were to *1*) develop an analysis method to evaluate membrane responses in this nonlinear domain and *2*) explore phase relationships between rhythmic stimuli and subthreshold and spiking membrane potential (V_memb_) responses in models of theta-resonant pyramidal neurons. Responses in these output regimes were asymmetrical, with different phase shifts during hyperpolarizing and depolarizing half-cycles. Suprathreshold theta-rhythmic stimuli produced nonstationary V_memb_ responses. Sinusoidal inputs produced “phase retreat”: action potentials occurred progressively later in cycles of the input stimulus, resulting from adaptation. Sinusoidal current with increasing amplitude over cycles produced “phase advance”: action potentials occurred progressively earlier. Phase retreat, phase advance, and subthreshold phase shifts were modulated by multiple ion channel conductances. Our results suggest differential responses of cortical neurons depending on the frequency of oscillatory input, which will play a role in neuronal responses to shifts in network state. We hypothesize that intrinsic cellular properties complement network properties and contribute to in vivo phase-shift phenomena such as phase precession, seen in place and grid cells, and phase roll, also observed in hippocampal CA1 neurons.

**NEW & NOTEWORTHY** We augmented electrical impedance analysis to characterize phase shifts between large-amplitude current stimuli and nonlinear, asymmetric membrane potential responses. We predict different frequency-dependent phase shifts in response excitation vs. inhibition, as well as shifts in spike timing over multiple input cycles, in theta-resonant pyramidal neurons. We hypothesize that these effects contribute to navigation-related phenomena such as phase precession and phase roll. Our neuron-level hypothesis complements, rather than falsifies, prior network-level explanations of these phenomena.

## Graphical abstract

**Figure:**
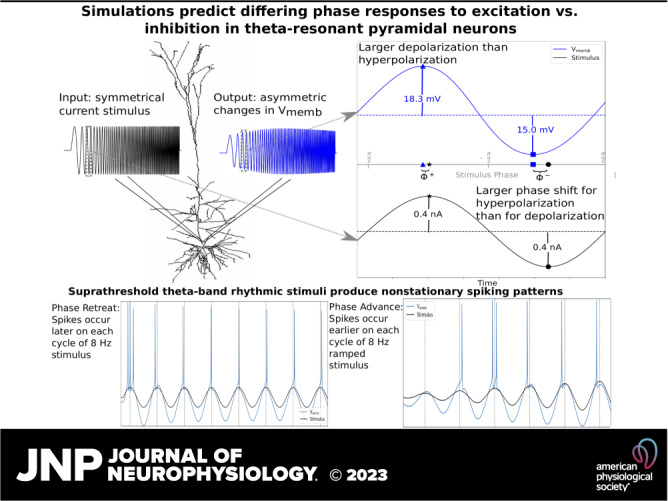

## INTRODUCTION

Rhythmic activity is ubiquitous in cortical structures of the brain and can be measured as oscillations in the local field potentials (LFPs) recorded extracellularly. LFPs are largely generated by transmembrane currents, principally postsynaptic dendritic currents, in neurons near the recording electrode (1, 2). Pyramidal neurons are among the largest contributors to these rhythms, particularly in the theta range near 8 Hz (3–5). Pyramidal neurons have diverse biophysics and projections, forming the main outputs of cortical circuits (6–9). Understanding how pyramidal neurons integrate oscillatory synaptic inputs to generate subthreshold membrane potential oscillations and spiking activity is therefore critical to understanding cortical computations (2, 10–13). Here, we focus on two types of pyramidal neurons: neocortical layer 5 b (L5b) pyramidal neurons and hippocampal CA1 neurons. L5b pyramidal neurons integrate inputs from the thalamus, other cortical layers, and other cortical areas and exert top-down control over subcortical brain areas (14–19). Hippocampal CA1 pyramidal neurons integrate rhythmic sensory-related inputs and encode “place” in an environment (20, 21). These cell types are theta-resonant: membrane potential (V_memb_) responds most strongly to subthreshold oscillatory inputs in the theta range of 4–10 Hz (22–25). Impedance analysis provides a tool for characterizing how the neuronal membrane responds to and filters oscillatory inputs.

The impedance analysis framework derives from electrical engineering and is based on linear RLC circuits composed of resistors (R), capacitors (C), and inductors (L). Impedance analysis assumes that the neuronal membrane is a linear, time-invariant (stationary) system, such as an RLC circuit. Linearity means that positive and negative current stimuli of equivalent amplitude will produce equal, opposite, and additive changes to V_memb_, typically not the case in a neural context. “Nonlinearity” of neuronal membrane response arises through voltage-gated ion channels, whose conductances are different during depolarization and hyperpolarization. Stationarity implies that the membrane always has the same response to equivalent inputs. “Nonstationarity” arises from intrinsic mechanisms that preserve history (history dependence) – slow channel kinetics, Ca^2+^ accumulation, neuromodulation (changing state or set-points), etc. Classical impedance analysis is therefore ill-suited for studying neuronal responses, which are markedly nonlinear and time varying (26, 27). Nonetheless, classical impedance analysis has been widely used for neuronal responses in a low-amplitude regime where the primary nonlinearity is approximated by a linear inductance, considered as a “phenomenological inductance” (28, 29).

Neuronal membrane impedance is usually estimated by applying current as either a chirp (time-varying signal whose instantaneous frequency increases with time) or white noise and recording the resulting changes in V_memb_ (30–32). Impedance is expressed as a complex number from which we can extract amplitude (|Z| – the frequency-dependent output strength) and phase (Φ – the frequency-dependent temporal shifts between corresponding points such as the peak or the trough in the input – the current *I* stimulus vs. that of the output – the V_memb_ waveform). Two important features of L5b and hippocampal pyramidal neuron impedance profiles are resonance (V_memb_ responds most strongly to an input frequency in the theta range) and synchrony (V_memb_ and a stimulus with matched timing). Resonance and synchrony are properties of inductive RLC circuits, as opposed to RC circuits such as a passive membrane, and are mediated in cortical pyramidal neurons by the phenomenological inductance generated by hyperpolarization-activated cyclic nucleotide-gated (HCN) ion channel currents (*I*_h_) or M-type K^+^ currents (*I*_M_) (22–24, 33–36). Because impedance methods properly pertain to a nonphysiological, linear, time-invariant/stationary system, we developed new methods for characterizing the nonlinear filtering properties of the neuronal membrane, whether spiking or subthreshold, demonstrating their implications for cellular contributions to oscillatory activity at the network scale.

A number of techniques have been used to expand the impedance framework to physiological signaling, with a focus on examining amplitude nonlinearities. For example, theta-frequency inputs were demonstrated to have an *I*_h_ -dependent nonlinearity in hippocampal pyramidal neurons (37), based on spike-triggered averaging (38). Ulrich (25) used chirp stimulation of neocortical L5b pyramidal neurons to produce “band-passed spiking”: action potentials were largely seen in response to frequencies around the cell’s subthreshold resonance frequencies. Asymmetric responses to excitation versus inhibition have also been noted (39–41). Pena et al. (42) extended the impedance amplitude framework to assess these asymmetries, identifying separate resonance frequencies for hyperpolarizing and depolarizing voltage responses. Similarly, Fischer et al. (43) identified the frequency dependence of leech-neuron spiking in response to sinusoidal inputs. With the focus on amplitude, temporal characteristics of nonlinear neural responses have been largely ignored.

In this paper, we devise and apply an analysis method to compute separate phase shifts for depolarization and hyperpolarization during nonlinear V_memb_ responses, both sub- and suprathreshold. We applied this method to three detailed models of theta-resonant pyramidal neurons from neocortical layer L5b and hippocampal CA1 (44–47) with the goal of better understanding their responses to rhythmic inputs. Nonlinear subthreshold V_memb_ responses to chirp stimuli produced different frequency-dependent phase relationships during depolarization and hyperpolarization which traditional impedance analysis did not capture. Chirp stimulation presented an incomplete picture of these relationships due to the nonstationarity of suprathreshold V_memb_ responses (phase shifts shifting over time). Action potentials occurred progressively later in the cycles of the input stimulus: “phase retreat.” Conversely, increasing stimulus amplitude on each cycle produced progressively earlier action potentials: “phase advance.” We suggest that these processes are involved in relationships between local field potential oscillations and spike timing observed in a number of brain structures in vivo and often ascribed to network phenomena (48–52): phase advance contributing to the phase precession seen in place cells, grid cells, frontal cortex, and other areas (53, 54); phase retreat contributing to phase roll in hippocampal CA1 neurons (55).

## METHODS

### Cell Models

In this study, we used three models of cortical pyramidal neurons: one neocortical layer 5 b (L5b) pyramidal neuron from rat (45, 46) (*model 1*), a L5b pyramidal neuron from mouse primary motor cortex (3, 46, 56) (*model 2*), and a hippocampal CA1 pyramidal neuron from rat (44, 47) (*model 3*).

*Model 1* included slow inactivating K^+^ current (57) and fast inactivating K^+^ current (57) expressed at the soma; fast inactivating Na^+^ current (58), persistent Na^+^ current (59), fast noninactivating K^+^ current [Kv3.1 (60)], small conductance Ca^2+^ activated K^+^ current [SK, *I*_AHP_ (61)] expressed at constant densities in the soma and dendrites, and muscarinic K^+^ current [*I*_M_ (62)] expressed at constant density throughout the dendrites. The model also included high voltage-activated (HVA) Ca^2+^ current [I*_HVA_* (63)] and low voltage-activated (LVA) Ca^2+^ current [I*_LVA_* T-type Ca^2+^ channel (64, 65)]. LVA and HVA Ca^2+^ channels were expressed most strongly in a “hot zone” between 685 and 885 mm from the soma, where their expression was 100 and 10 times higher, respectively, than elsewhere in the dendrites or soma, where their density was constant. Equations governing the spatial distributions and kinetics of these channels are fully described by Hay et al. (45). The original model included a nonspecific cation current [HCN channel, *I*_h_ (66)] expressed in constant density in the soma and basal dendrites and with increasing density with distance from the soma in the apical dendrites. We replaced the original model of *I*_h_ with a combination of *I*_h_ and a Twik-associate acid-sensitive K^+^ (TASK)-like shunting current as described by Migliore and Migliore (47). The expression of *I*_h_ and TASK-like shunting current followed the same distribution as *I*_h_ in the original model. Previous work demonstrated that this version of the model, unlike the original or a number of other detailed models of L5b pyramidal neurons, exhibited realistic location-dependent subthreshold impedance properties (e.g., resonance frequency, synchronous frequency) (46) and that motivated its use in this study. Unless otherwise noted, simulations in this study were run using this model.

*Model 2* differed from *model 1* in morphology and in ion channel types and distributions. Similar to *model 1*, *model 2* included a combination of *I*_h_ and a TASK-like shunting current as described by Migliore and Migliore (47) and exhibited realistic location-dependent subthreshold impedance properties (46). Unlike *model 1*, the expression of *I*_h_ and TASK-like shunting current saturated in the apical tufts rather than continuing to increase exponentially. It also included a hot zone of LVA and HVA Ca^2+^ currents in the apical dendrite, fast inactivating Na^+^ current (58), persistent Na^+^ current (59), small conductance Ca^2+^ activated K^+^ current [SK, *I*_AHP_ (61)] expressed at constant densities in the soma and dendrites, and muscarinic K^+^ current [*I*_M_ (62)] expressed at constant density throughout the dendrites. Furthermore, *model 2* included A-type K^+^ currents (*I*_A_) expressed at constant density in the dendrites and soma, which was absent in *model 1*. Further details about the model, its ion channels, and their distributions may be found in studies by Neymotin et al. (56), Dura-Bernal et al. (3), and Kelley et al. (46).

*Model 3* again had its own morphology, ion channels, and distributions thereof. Like the previous two models, it included a combination of *I*_h_ and a TASK-like shunting current as described by Migliore and Migliore (47), and as in model 2, expression of these currents saturated in the apical tufts. It also included A-type K^+^ currents (*I*_A)_ in the soma and dendrites (67), fast inactivating Na^+^ current in the axon, persistent Na^+^ current in the soma and dendrites, and M-type K^+^ currents (*I*_M)_ expressed in the soma and axon (68). Further details about the model can be found in studies by Ascoli et al. (44) and Migliore and Migliore (47).

### Simulations and Analysis

As previously described (31), a linear chirp current stimulus was defined as:

(*1*) Iin(t)=A  sin[2π(c2t2+f0t)],

where *c* = (*f*_1_–*f*_0_)/*T*, *f*_0_ is the initial frequency in Hz, *f*_1_ is the final frequency, and *T* is the duration of the frequency sweep in seconds (*f*_1_ = 0.5; *f*_2_ = 12; *T* = 12). The stimulus amplitude, *A*, was chosen to be either subthreshold linear, subthreshold nonlinear, or suprathreshold with 1 spike/cycle at low frequency (≤12 Hz).

**Figure 1.**
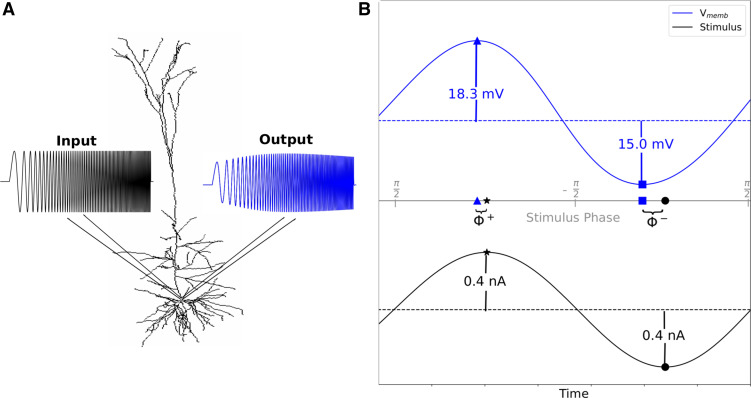
High amplitude stimuli produced nonlinear V_memb_ responses with different phase shifts for depolarization and hyperpolarization. *A*: morphology of L5b neuron (*model 1*) with input signal, symmetric chirp current stimulus (black), and output signal, asymmetric changes in V_memb_. *B*: single cycle of symmetrical, sinusoidal current stimulus (black, *bottom*), which produced asymmetric V_memb_ response (blue, *top*). Superscripted plus (^+^) and minus (^–^), respectively, label depolarizing and hyperpolarizing half-cycle peaks and troughs. Times of the V_memb_ peaks and troughs were mapped onto instantaneous stimulus phase. Difference in instantaneous stimulus phase between time of peak V_memb_ to peak in stimulus (triangle to star, Φ^+^) or trough to trough (square to circle Φ^–^) was the value of the phase shift: here, Φ^+^ = 0.13, Φ^–^ = 0.40 for a 3 Hz input. Both are positive, indicating phase lead (V_memb_ peak preceding stimulus peak) for both. V_memb_, membrane potential.

Since impedance analysis is inadequate for asymmetric, nonlinear responses, we extended a framework for computing a nonlinear analog of impedance amplitude developed by Pena et al. (42) to compute a nonlinear analog of impedance phase (Φ) for depolarization and hyperpolarization. We defined the upper (+) and lower (−) envelopes of V_memb_ and *I_in_* by finding the peaks ($V_{n}^{+}$) and troughs ($V_{n}^{-}$) in V_memb_ and the peaks/troughs in the stimulus ($I_{n}^{\pm }$) for each stimulus cycle *n* (Fig. 1). We defined the times of each peak/trough in V_memb_ as $v_{n}^{\pm }$ and the times of each peak/trough in the stimulus as $i_{n}^{\pm }$. The instantaneous phase [*P*(*t*)] of the current stimulus was extracted using the Hilbert transform:

(*2*) $$P(t)=∠Hilbert[I_{in}(t)].$$

We defined our nonlinear Φ analog for the upper and lower V_memb_ envelopes (+/−, respectively) as:

(*3*) $$\Phi_{n}^{\pm }=\{\begin{array}{ll} P(i_{n}^{\pm })-P(v_{n}^{\pm }), & \text{if }P(v_{n}^{\pm })>0\\ P(i_{n}^{\pm })-(P(v_{n}^{\pm })+2\pi ), & \text{otherwise} \end{array}\;\;n=0,1,2,\ldots ,N,$$

where *N* is the total number of stimulus cycles; the 2π correction was required when a V_memb_ trough *v*^−^ occurred after a stimulus trough *i*^–^, thus on the following cycle of *P*(*t*). Since the chirp stimulus has an instantaneous frequency that increases linearly with time, one can readily define $\Phi^{\pm }$(*f*) where *f* is stimulus frequency, analogously with $\Phi_{n}^{\pm }$. As with linear Φ, phase lead occurs when $\Phi_{n}^{\pm }>0$: peaks/troughs of V_memb_ preceding peaks/troughs of *I_in_*(*t*). Similarly, phase lag is defined as $\Phi_{n}^{\pm }<0$, where peaks/troughs in V_memb_ lag behind the input stimulus. We defined a “synchrony point” as a stimulus frequency where $\Phi_{n}^{\pm }=0$ representing a transition point between lead and lag where peaks/troughs in V_memb_ and stimulus are synchronous. We computed an input $\Phi_{n}^{\pm }(f)$, stimulating and recording V_memb_ at the soma, for chirp stimuli in subthreshold, suprathreshold, and spike-blockade suprathreshold conditions, comparing to the near-linear response to small subthreshold chirp, calculated using Fourier-based methods. For the suprathreshold spiking conditions, we computed Φ^+^ of a single spike or the first spike in a burst. We also computed transfer $\Phi_{n}^{\pm }$, stimulating the dendrite and recording V_memb_ and the soma.

Because responses to suprathreshold chirp stimuli combined the effects of frequency shift with nonstationary cell response, we switched to regular sinusoidal stimuli to assess $\Phi_{n}^{+}$ across cycles of constant frequency of 2–9 Hz. We also assessed sinusoidal stimuli of increasing amplitude: starting at 0 nA with a linear increase of 0.32 nA/s unless noted otherwise.

In the course of this study, we ran more than 700 single-cell simulations. One second of simulation time using *model 1* required ∼10.5 s of clock-time in NEURON on a Linux system using a single core of a 1.60 GHz Intel Core i5-10210U CPU. All simulations presented here were run using NEURON version 8.0 (69). The code developed for simulation, data analysis, and visualization was written in Python, and it is available https://github.com/suny-downstate-medical-center/nonlinear_impedance_phase.

## RESULTS

**Figure 2.**
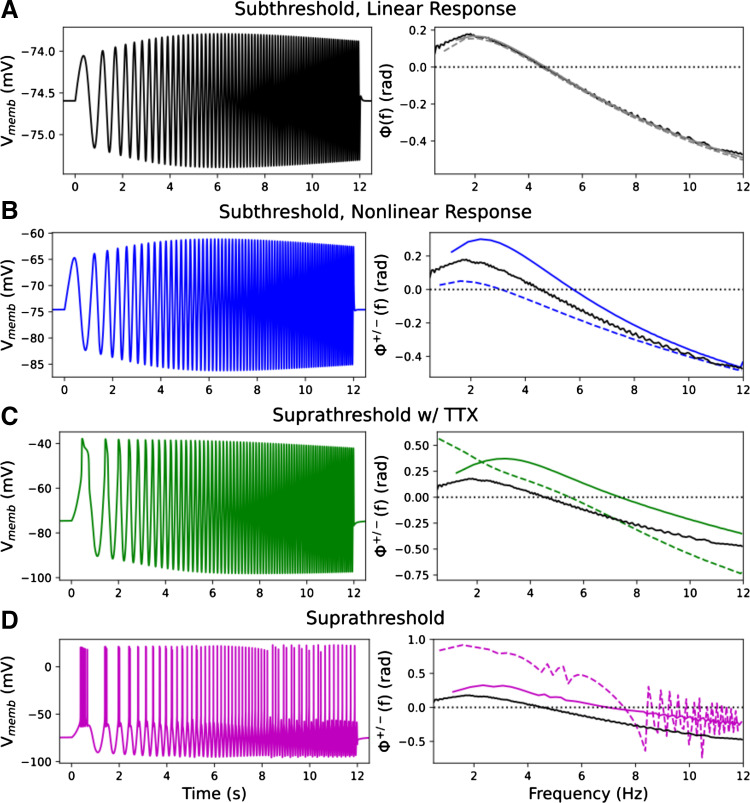
Higher amplitude chirp stimuli (input, I) produced nonlinear V_memb_ responses (output, O) with differing frequency-dependent I/O phase relationships for depolarization vs. hyperpolarization. [*Left*: V_memb_, *right*: Φ^±^(*f*); Φ^+^(*f*) depolarizing: dashed lines, Φ^–^(*f*) hyperpolarizing: solid lines; Φ^±^(*f*) > 0 means V_memb_ peak/trough *leads* (precedes) stimulus peak/trough; Φ^±^(*f*) = 0 means V_memb_ peak/trough and stimulus peak/trough were synchronous; Φ^±^(*f*) < 0 means V_memb_ peak/trough *lags* (follows) stimulus peak/trough.] *A*: small amplitude stimulus produced characteristic near-linear response [Φ^+^(*f*) and Φ^–^(*f*) overlaid in gray]. *B*: higher amplitude subthreshold stimulus showed mildly nonlinear amplitude response (peak 5% larger than trough) and differences in phase response (black line reproduced from *A* in *B*–*D* for reference). Resonance frequencies for depolarization and hyperpolarization were similar (6.4 and 6.6 Hz, respectively; Supplemental Fig. S1). *C*: suprathreshold chirp with blocked spikes (simulated TTX experiment). Amplitude and phase differences are more pronounced than in *B*. *D*: spiking patterns (dashed line) showed still greater phase lead. Hyperpolarizing responses were similar to those in *C*. TTX, tetrodotoxin; V_memb_, membrane potential.

Traditionally, impedance measures used for characterizing neuronal input/output (I/O; current/V_memb_) relationships relied on low-amplitude signals below the thresholds for activation of most voltage-sensitive ion channels (31, 70). This had the advantage of providing a near-linear I/O relationship that could be characterized using traditional electrical engineering methods. However, a major disadvantage was that it excluded larger, more physiologically-relevant signals with amplitudes comparable to compound postsynaptic potentials. To characterize the time course of neuronal responses to oscillatory stimuli in this physiological regime, we provided higher amplitude inputs than typically used, high enough to activate voltage-sensitive ion channels. We then separated the phase measures for depolarizing peaks (Φ^+^) and hyperpolarizing troughs (Φ^–^), using the alternating half-cycles of the inputs (see methods). These two phase measures were found to characterize very different I/O phase relationships, assessed both with and without spiking activity (Fig. 2). Evidence of nonstationarity in the phase relationship between spiking activity and stimulus (change in phase shift between spikes and stimulus over time), led us to assess responses to more prolonged fixed-frequency sinusoids in addition to chirps. Identified patterns demonstrated how the intrinsic electrophysiology of a single neuron could give rise to the theta-band phase shifts seen in behaving animals: phase precession (54) and phase roll (55). Unless noted otherwise, simulations were performed in *model 1* (45, 46) (see methods for model difference details).

As previously demonstrated experimentally in L5b pyramidal neurons (23, 25, 71) and in models (46), small, subthreshold chirp stimuli produced near-linear responses (Fig. 2*A*). Φ^−^(*f*) and Φ^+^(*f*) are nearly identical – the traditional unitary impedance phase. *I*_h_ produced “phenomenological inductance,” where the neuronal membrane, rather than behaving like a low-pass (RC) circuit, produced resonance and other properties resembling a band-pass (RLC) circuit, without employing an actual inductor. Due to the phenomenological inductance generated by *I*_h,_ input and output were in phase at the “synchrony point” (Φ = 0; here at 4.5 Hz), and there was inductive phase or phase lead (Φ > 0; output leads input) at frequencies below the synchrony point. There was phase lag (Φ < 0; output lags behind input) at frequencies higher than the synchrony point (Fig. 2*A*). For higher amplitude stimuli producing nonlinear V_memb_ response (maximum peak 5% larger than minimum trough), we analyzed the upper and lower envelopes of V_memb_ separately and found that they showed different frequency-dependent I/O phase relationships [Fig. 2, *B*–*D*; Φ^+^(*f*) – depolarizing, dashed; Φ^–^(*f*) – hyperpolarizing, solid line]. Compared with the low-amplitude responses (black lines in Fig. 2, *B*–*D* are copied from Fig. 2*A* for comparison), high-amplitude subthreshold stimuli produced less phase lead and a lower synchrony point (3 Hz) during depolarizing half-cycles (dashed blue), but they produced more phase lead and a higher synchrony point (6 Hz) during hyperpolarizing half-cycles (solid blue). We also computed impedance amplitude differences for depolarizing and hyperpolarizing half-cycles [as in Pena et al. (42]; the resonance frequencies were nearly indistinguishable (6.4 and 6.6 Hz, respectively; Supplemental Fig. S1). Thus, these additional nonlinearities substantially altered timing of depolarization versus hyperpolarization response with little change in frequency selectivity. For suprathreshold stimuli with spiking blocked, simulating a tetrodotoxin (TTX) experiment, both depolarizing and hyperpolarizing responses showed more phase lead than seen with either subthreshold example (Fig. 2*C*; note broadened *y* axis); synchrony points were both shifted to higher frequencies: 5.5 and 7.5 Hz, respectively.

As expected, spiking provided a major change in phase relationships for the depolarizations, with little change in phase relationships for hyperpolarizations (Fig. 2*D*). The initial burst and subsequent spikes occurred during the upswings of the input wave where the voltage reached a threshold. The spike is itself a rapid depolarization that easily beats the low-frequency initial cycle of chirp stimulus to the peak, providing the large phase lead of almost 1 radian. This phase lead continued at higher frequencies up to the synchrony point of 7.5 Hz, higher than in the other cases, solidly in the theta band. For stimulus frequencies above 8.3 Hz, spikes did not occur on every stimulus cycle, giving the varying phase shift over successive cycles, alternately preceding or following the stimulus peak (Fig. 2*D*, purple dashed line).

**Figure 3.**
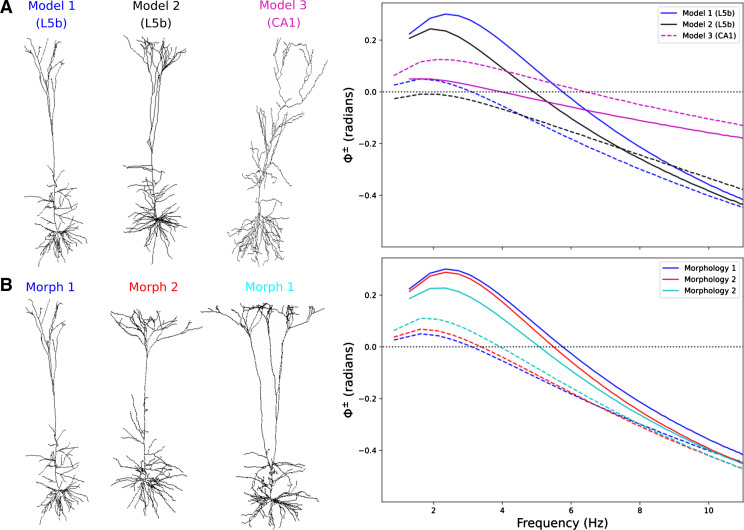
Different models of cortical pyramidal neurons exhibited different phase shifts for depolarization and hyperpolarization. *A*: morphologies (*left*) of 3 models of cortical pyramidal neurons (two from L5b, one from hippocampal CA1) and their frequency-dependent phase shifts for depolarization (dashed lines) and hyperpolarization (solid) in response to high amplitude subthreshold chirp stimuli (*right*). Each cell model has a unique channel distribution (see methods; *Model 1* curves are same as in Fig. 2*B*). *B*: morphologies (*left*) of 3 L5b pyramidal neuron models with the same channel distributions (from *model 1*) and their frequency-dependent phase shifts for depolarization (dashed lines) and hyperpolarization (solid) in response to high amplitude subthreshold chirp stimuli (*right*). Morphology 1 is used in *model 1*.

High amplitude subthreshold chirp stimuli produced different frequency-dependent phase shifts for depolarization and hyperpolarization in 3 different theta-resonant pyramidal neuron models (Fig. 3*A*). *Models 1* and *2* are models of L5b pyramidal neurons with different morphologies and different channel distributions; of these, *model 2* exhibited lower synchronous frequency and less phase lead for hyperpolarization and no synchrony or phase lead for depolarization. *Model 3*, a model of a hippocampal CA1 pyramidal cell, exhibited phase lead and synchrony for depolarization and hyperpolarization, like *model 1*, but with greater phase lead and higher synchrony point for depolarization than hyperpolarization, unlike in the other models. We also investigated the role of morphology by using the channel distribution from *model 1* with different L5b pyramidal neuron morphologies from neuroanatomical tracings (Fig. 3*B*). Morphology’s effect on phase shifts was far less than that seen across the different models in Fig. 3*A*.

**Figure 4.**
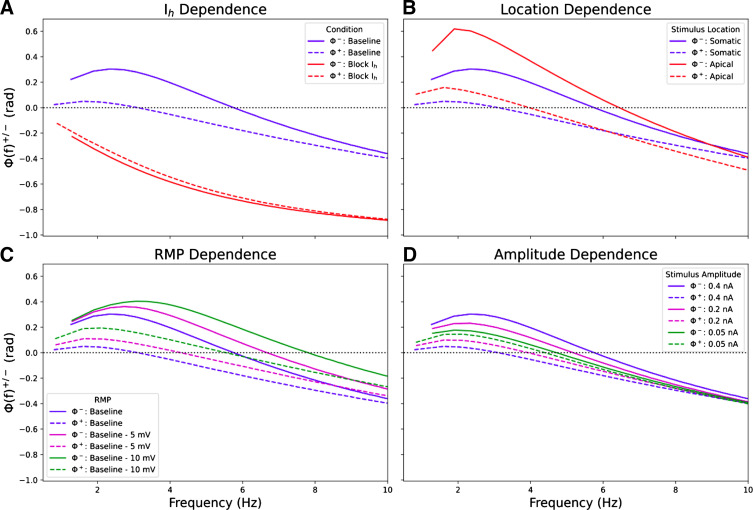
I/O phase shifts in subthreshold, nonlinear regime depended on *I*_h_, and stimulus parameters and location. Φ^+^(*f*) (dashed lines) and Φ^–^(*f*) (solid lines) when blocking *I*_h_ vs. control (*A*), when stimulating the apical dendrite (260 μm from the soma) vs. the soma (*B*), under different artificially set RMPs (*C*), and for different stimulus amplitudes (*D*). Phase shifts for depolarization and hyperpolarization were also sensitive to changes in the activation time constant for *I*_h_ (Supplemental Fig. S2.). I, input; O, output; RMP, resting membrane potential.

In the models of L5b pyramidal neurons, I/O phase relationships for both hyperpolarizing and depolarizing responses in the high-subthreshold regime were dependent on the phenomenological inductance produced by *I*_h_ (Fig. 4). Blocking *I*_h_ (simulating ZD7288 application) eliminated inductive phase shift, synchrony between stimulus and V_memb_, and phase differences for hyperpolarization and depolarization (Fig. 4*A*) while retaining V_memb_ amplitude difference (peak 5% higher than trough) due to the effect of *I*_Naf_. Stimulus location altered phase due to the higher *I*_h_ density in the dendrites compared with the soma (Fig. 4*B*). Stimulating in the apical dendrite (260 μm from the soma) produced increased phase lead and higher synchrony points for both depolarization and hyperpolarization compared with stimulating the soma. Because *I*_h_ activation is voltage-dependent, the phase was altered by changes in resting membrane potential (RMP) and stimulus amplitude. RMP decrement by constant negative current stimulus brought the voltages closer to the *I*_h_ half-activation point so that hyperpolarizing responses were increased at decreased RMP (−5 and −10 mV from baseline of −74 mV) and increased activation of *I*_h_ increased phenomenological inductance (29, 70). Decreasing RMP by 10 mV increased phase lead and the synchrony points (by 2.1 Hz for hyperpolarization and 2.5 Hz for depolarization) (Fig. 4*C*). Changes in stimulus amplitude affected the degree to which different voltage-gated ion channels open (namely, HCN and voltage-gated Na^+^ channels) and thus the degree of nonlinearity in the V_memb_ response. Very small (0.05 nA) stimuli produced a near-linear response similar to Fig. 2*A*, with negligible difference for depolarization and hyperpolarization (Fig. 4*D*, green lines). With increasing stimulus amplitude, I/O phase relationships for hyperpolarization and depolarization diverge from the traditional impedance phase [Φ(*f*)]: differences in the synchrony points were caused by regenerative effects involving *I*_h_ for hyperpolarization and *I*_Naf_ for depolarization (Fig. 4*D*).

**Figure 5.**
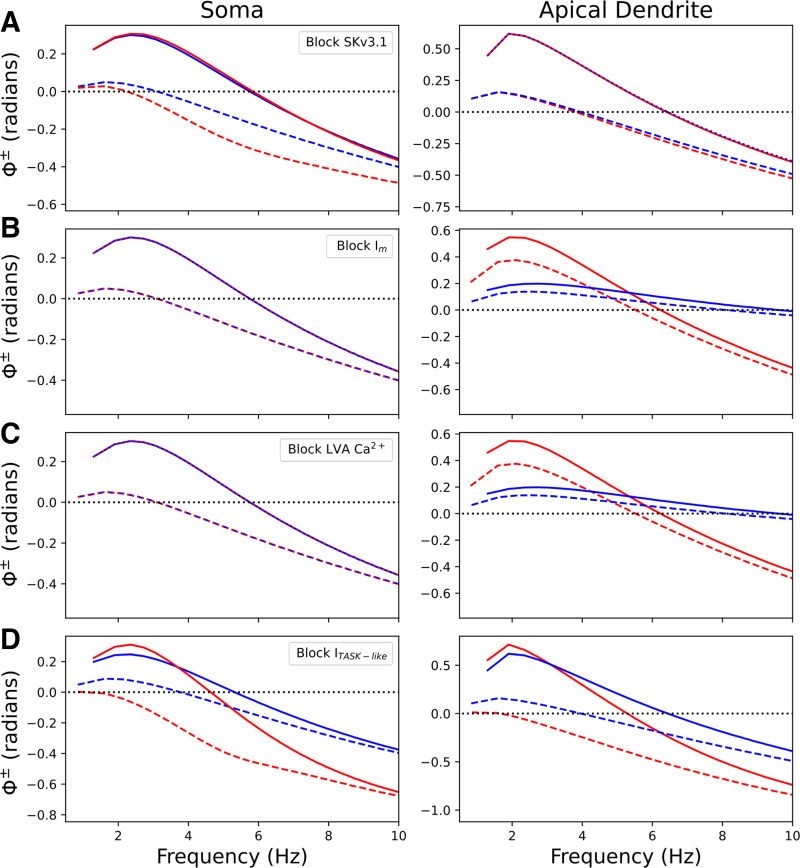
Multiple channels produce phenomenological inductance. Phase shifts for hyperpolarization (solid) and depolarization (dashed) at soma (*left*) and apical dendrite (*right*) for control (blue) and channel block (red). Note differences in *y* axis across panels. *A*: blocking SKv3.1 channels modulated depolarization phase shifts at soma. *B*: blocking *I*_M_ modulated both phase shifts for stimulation at apical dendrite (260 μm out from soma). *C*: blocking LVA Ca^2+^ channels modulated both phase shifts for stimulation of apical dendrite near Ca^2+^ “hot-zone” (500 μm out). *D*: blocking TASK-like shunting current modulated both phase shifts at both the soma and in the apical dendrite (260 μm out). Blocking TASK-like shunting current increased excitability, requiring the use of a lower amplitude stimulus. We also simulated *I*_A_ blockade (*I*_A_ contained in *Model 2* only) (Supplemental Fig. S3). LVA, low voltage-activated.

Previous work demonstrated that the phenomenological inductance generated by *I*_h_ was modulated by other subthreshold currents such as *I*_A,_ T-type Ca^2+^ current, and TASK-like shunting current (46, 72, 73). Our simulations predict location-dependent modulation of I/O phase shifts by a number of different currents (Fig. 5). Blocking Skv3.1 channels modulated the phase shifts for depolarization, not hyperpolarization, at the soma (Fig. 5*A*). This resulted in less phase lead for depolarization. Blocking Skv3.1 channels had no influence on phase shifts in the apical dendrite. Conversely, blocking *I*_M_ and LVA Ca^2+^ channels modulated phase shifts for both depolarization and hyperpolarization in the apical dendrites (500 μm from the soma), but not at the soma or closer along the apical dendrite (260 μm away) (Fig. 5, *B* and *C*). Blocking either *I*_M_ or LVA Ca^2+^ channels had nearly identical effects on phase shifts in the apical dendrite, increasing phenomenological inductance and phase lead. Blocking the TASK-like shunting current affected I/O phase shifts both at the soma and in the apical dendrite, reducing phenomenological inductance and phase lead (Fig. 5*D*). Blocking the TASK-like shunting current also increased membrane excitability, so smaller amplitude stimuli (0.25 nA at the soma, 0.3 nA in the apical dendrite) were used to prevent spiking. As model 1 did not include *I*_A,_ we simulated *I*_A_ blockade in model 2 (Supplemental Fig. S3). Blocking *I*_A_ modulated phase shifts more strongly for depolarization than hyperpolarization and more strongly at the soma than in the apical dendrite. The location dependence of these ion channel modulations of I/O phase shifts was in line with differences in their distributions.

**Figure 6.**
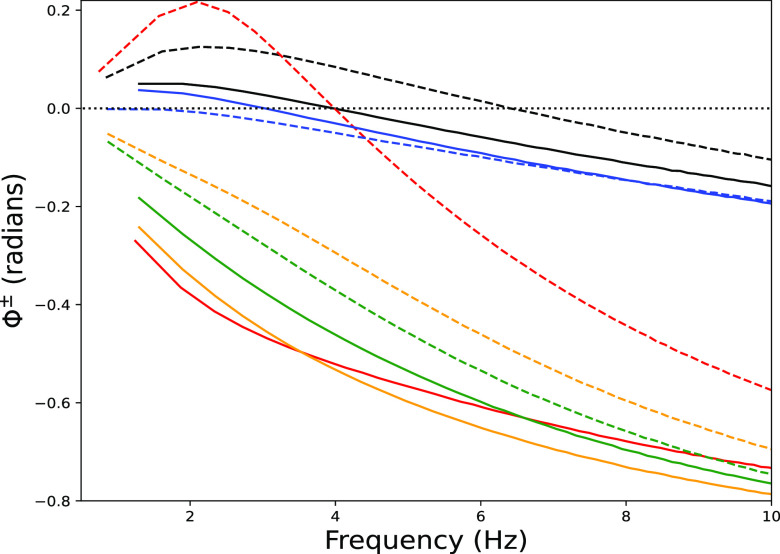
Multiple resonant currents contributed to phenomenological inductance in the hippocampal CA1 pyramidal neuron model. High-amplitude subthreshold chirp stimuli under control conditions and with channel blockade. Blocking *I*_h_ (red) eliminated phase lead and synchrony between stimulus and V_memb_ for hyperpolarization (solid) but preserved *I*_M_-dependent phenomenological inductance that produced phase lead and synchrony for depolarization (dashed). Blocking *I*_M_ (blue) preserved phase lead and synchrony for hyperpolarization, not depolarization. Blocking both currents eliminated phenomenological inductance. Blocking both and *I*_A_ (green) reduced differences between Φ^+^ and Φ^–^. V_memb_, membrane potential.

The dependence of I/O phase shifts on channel conductances was still more complex in the model of a hippocampal CA1 pyramidal neuron (*model 3*; Fig. 6). In this cell, as opposed to the models of L5b cortical pyramidal neurons, phenomenological inductance was generated by both *I*_h_ and *I*_M,_ both expressed strongly in this cell type. *Model 3* also showed higher Φ^+^ than Φ^–^, reversing the pattern previously seen in the models of L5b pyramidal neurons. Blocking *I*_h_ eliminated synchrony and phase lead for hyperpolarization but not for depolarization. This is because *I*_M_ generates its own perithreshold phenomenological inductance (22, 33, 34, 36, 74) alongside *I*_h_, which produced resonance and here selectively increased phenomenological inductance for high amplitude depolarizations. Blocking *I*_M_ preserved the *I*_h_-dependent phenomenological inductance, producing phase lead for hyperpolarization and phase lag for depolarization across stimulus frequencies. Blocking both *I*_h_ and *I*_M_ eliminated phenomenological inductance, and therefore phase lead and synchrony for both depolarization and hyperpolarization, but there remained a large separation between Φ^+^ and Φ^–^. This separation was narrowed by also blocking *I*_A_.

**Figure 7.**
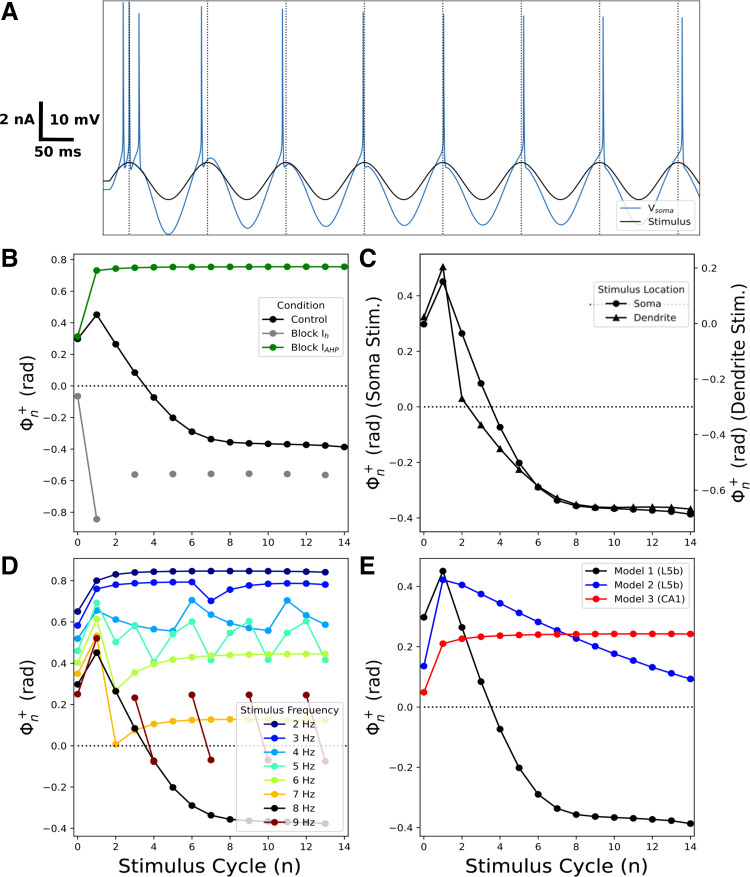
Sinusoidal stimuli produced phase retreat. *A*: 8 Hz sinusoidal 1 nA current stimulus (black) in soma; V_memb_ in blue – distance from 0-radian marks (vertical lines) to spike increased on each cycle. *B*: phase retreat was eliminated by blocking *I*_h_ (gray) or *I*_AHP_ (green), and modulated by blocking other currents (Supplemental Fig. S4, *left*) or by changing current amplitude (Supplemental Fig. S5*A*). *C*: location dependence (note dual *y* axis to compensate for signal delay from dendrite; dendritic stimulation increased to provide similar spiking at soma). *D*: frequency dependence. *E*: differences across the 3 models. V_memb_, membrane potential.

We examined phase shifts for spiking during a constant-frequency sinusoidal stimulus to determine how they changed with the stimulus cycle, *n*. For particular combinations of stimulus amplitude and frequency, $\Phi_{n}^{+}$ decreased over the course of multiple stimulus cycles: “phase retreat” (Fig. 7*A*). Using an 8 Hz stimulus, the first spike (part of a burst) occurred before the peak of the first stimulus cycle (*n* = 0). $\Phi_{n}^{+}$ then decreased over subsequent cycles due to adaptation. Spikes occurred after the stimulus peak from the fourth stimulus cycle onward (*n* = 4) until reaching a steady state above *n* = 8 which $\Phi_{n}^{+}$ changed little. Thus, during phase retreat, $\Phi_{n}^{+}$ encoded the duration of the stimulus before reaching a steady state. The same stimulus also produced phase retreat in *model 2*, albeit over a smaller range of Φ^+^ than in *model 1* (Fig. 7*E*). Phase retreat was primarily due to *I*_h_ and *I*_AHP_ (SK channels) (Fig. 7*B*), as *I*_h_ inactivated and *I*_AHP_ activated due to depolarization and subsequent Ca^2+^ entry (SK is a Ca^2+^-dependent K^+^ channel). Blocking *I*_h_ or *I*_AHP_ eliminated changes in $\Phi_{n}^{+}$ over multiple cycles, eliminating retreat. Phase retreat also occurred when stimulating the apical dendrite (Fig. 7*C*), with the phase curve shifted downward (more delayed) due to conduction delay from the apical dendrite to soma. Differences in phase shift across the early cycles (2–4) were due to channel differences in the apical dendrite compared with soma. All of the currents that modulated phenomenological inductance in the subthreshold regime modulated Φ^+^ to varying degrees during phase retreat (Supplemental Fig. S4, *left*). Increasing stimulus amplitudes produced less phase retreat as the threshold on each cycle was reached before the current peaked (Supplemental Fig. S5A). For lower amplitudes (0.9 nA), the cell could not follow, producing dropped spikes with corresponding discontinuous phase relation. Phase retreat was frequency-dependent since low frequencies did not produce significant adaptation and in fact provided a slight facilitatory effect (Fig. 7*D*). Phase retreat was most pronounced at the theta frequency of 8 Hz, the fastest following frequency for this stimulus amplitude. At 9 Hz the cell could not follow, again producing dropped spikes. Our neocortical cells (*models 1* and *2*) exhibited phase retreat, but the hippocampal CA1 cell (*model 3*) did not, due to a lack of *I*_AHP_ (SK channels) with a consequent lack of spike adaptation (Fig. 7*E*).

**Figure 8.**
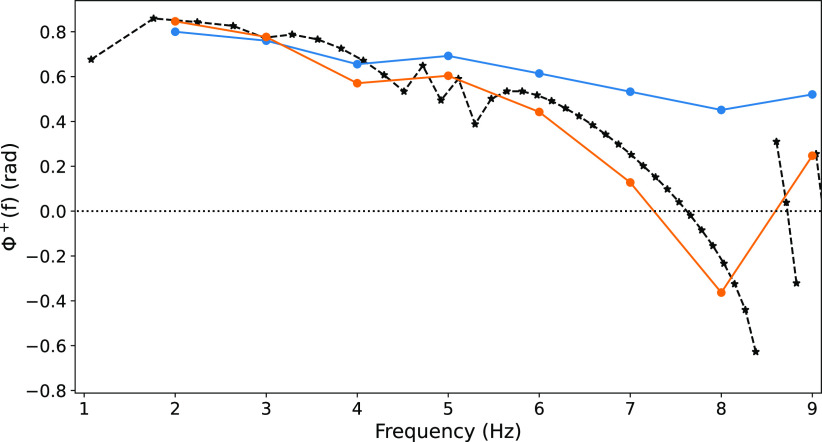
Suprathreshold chirp stimulation predicted steady-state $\Phi_{n}^{+}$. $\Phi_{n}^{+}$ for *n* = 1 (blue), *n* = 9 (orange) compared with Φ^+^(*f*) from chirp (black, reproduced from Fig. 2*D*).

We examined whether chirp stimulus could predict this nonstationarity spiking response (Fig. 8). Prediction was strong for the steady-state (adapted) depolarization phase [$\Phi_{n=9}^{+}$ compared with chirp Φ^+^(*f*)], while the phase shift during earlier stimulus cycles ($\Phi_{n=1}^{+}$) was not captured except at low frequencies where little change in phase took place (Fig. 7*D*, 2 and 3 Hz).

**Figure 9.**
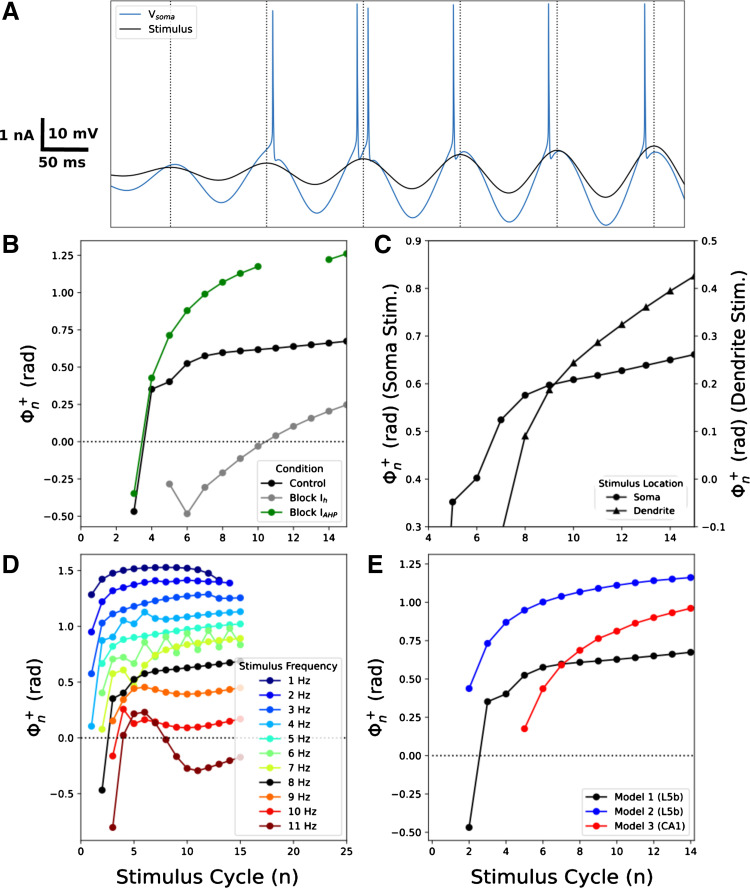
Increasing stimulus amplitude over time produced phase advance. *A*: 8 Hz sinusoidal current stimuli with amplitude increasing from 0.11 nA at 0.04 nA/cycle (black) in soma; V_memb_ in blue – distance from 0-radian marks (vertical lines) to spike shifted on each cycle. *B*: blocking *I*_h_ and *I*_AHP_ modulated phase advance, as did blockade of other currents (Supplemental Fig. S4, *right*). The rate of amplitude increase had little effect (Supplemental Fig. S5*B*). *C*: location dependence: phase advance was stronger when stimulating the apical dendrite (260 μm from the soma; dual *y* axis to compensate for signal delay from dendrite). *D*: frequency dependence (amplitude increase here 0.32 nA/s). *E*: differences across the 3 models. Due to the strong influence of *I*_M_ on subthreshold phase shifts in the CA1 pyramidal cell model, we simulated blocking *I*_M_ during phase advance (Supplemental Fig. S7*B*).

We simulated increased input due to synaptic facilitation or input augmentation (see discussion of place cell precession) by linearly increasing amplitude during the sinusoidal stimuli (Fig. 9). As expected, $\Phi_{n}^{+}$ increased over the course of multiple stimulus cycles as the cell reached spike threshold earlier in the stimulus cycle than on the previous: “phase advance.” $\Phi_{n}^{+}$ increased on each cycle, with phase lag on the first cycle and phase lead on later cycles (Fig. 9*A*). Spike timing was not just due to firing at the same stimulus amplitude each time but was instead associated with the time of progressively higher stimulus amplitudes, due to the interplay of adaptation and augmenting stimulation (Supplemental Fig. S6). Encoding of stimulus amplitude/duration only lasted for five stimulus cycles before $\Phi_{n}^{+}$ reached steady state in baseline conditions (Fig. 9*A*). [Phase advance was also seen in *model 2* over a smaller range of Φ^+^ and with higher average $\frac{d\Phi_{n}^{+}}{dn}$ (Fig. 9*E*).] Rate of stimulus amplitude change had relatively little influence on Φ^+^ (Supplemental Fig. S5*B*). Phase advance was modulated in opposing directions by *I*_h_ versus *I*_AHP_ blockade (Fig. 9*B*). Blocking *I*_AHP_ enhanced phase advance by increasing the range of $\Phi_{n}^{+}$ and increasing $\frac{d\Phi_{n}^{+}}{dn}$ for more stimulus cycles. However, there was spike failure on cycles 11–13 (Fig. 9*B*, interruption in green curve). Blocking *I*_h_ also augmented phase advance, increasing $\frac{d\Phi_{n}^{+}}{dn}$ to a similar extent. Differences in phase shift across the early cycles (2–4) were due to channel differences in the apical dendrite compared with soma. All of the currents that modulated phenomenological inductance in the subthreshold regime modulated $\Phi_{n}^{+}$ to varying degrees during phase advance (Supplemental Fig. S4, *right*). Phase advance was also location dependent (Fig. 9*C*; note elimination of delay due to location), with increased $\frac{d\Phi_{n}^{+}}{dn}$ produced by increased influence of ion channels in apical dendrite. Phase advance was strongly frequency-dependent (Fig. 9*D*). All three models produced phase advance in response to the theta-band 8 Hz ramped stimulus (Fig. 9*E*). Blocking *I*_M,_ which had a strong influence on subthreshold phase shifts, modulated phase advance in the model of hippocampal CA1 pyramidal cell (Supplemental Fig. S7*B*).

## DISCUSSION

Classical impedance analysis in neurophysiology requires low-amplitude signals to remain close to a linear ideal: the resistor-inductor-capacitor (RLC) circuit (Fig. 2*A*; 28, 70). These small signals only slightly activate voltage-sensitive ion channels, giving rise to a phenomenological inductance which can then be further analyzed using traditional electrical engineering techniques. However, small-signal inputs do not help us understand the strongly nonlinear responses from the far higher amplitude signals characteristic of neural inputs. In this paper, we presented an analysis method to compute the varying I/O phase shifts across larger-amplitude stimulus responses that are not seen using traditional impedance analysis. We identified different frequency-dependent phase shifts for subthreshold depolarization and hyperpolarization, suggesting different phase shifts for responses to inhibitory versus excitatory postsynaptic potentials. These results are important for understanding the response of neurons to the incoming oscillatory inputs that create field potential oscillations, particularly in the theta band.

We also used our analysis method to characterize nonstationary spiking responses. We hypothesize that these nonstationary cellular I/O phase relationships contribute to in vivo phase relationships between spiking and LFP oscillations related to navigation and cognition. The specific results presented here showed generalization across the neuronal models we used—all models of theta-resonant pyramidal neurons. However, in any particular neuron, the intrinsic parameters of spiking will also contribute, determining, for example, the tendency of a neuron to burst rather than produce a single spike. Future experiments could use higher amplitude chirp stimuli, along with suprathreshold sinusoidal stimuli, to produce nonlinear subthreshold V_memb_ responses and gain a more comprehensive understanding of I/O properties of neurons. Data from such experiments could in turn be used in the development of increasingly sophisticated neuronal models.

We defined the “synchrony point” as the crossover frequency from phase lead to phase lag where input and output are in phase (Φ^±^ = 0). Classical low-amplitude signal responses produced very similar phase relationships for hyperpolarizing and depolarizing half-cycles, sharing a single synchrony point (Fig. 2*A*). Higher amplitude subthreshold stimuli produced V_memb_ responses with different phase shifts and different synchrony points for depolarization and hyperpolarization (Fig. 1 and Fig. 2*B*). The models of L5b pyramidal neurons (*models 1* and *2*) showed more phase lead and a higher synchrony point for hyperpolarization compared with depolarization due to increased *I*_h_ (Fig. 2*B* and Fig. 3*A*). Phase lead and synchrony were dependent on the phenomenological inductance generated by *I*_h_ and its interactions with different voltage-gated ion channels activated during stimulation (Fig. 4). Blocking *I*_h_ eliminated the phenomenological inductance and effectively abolished the differences between the I/O phase relationships for depolarization and hyperpolarization, without however abolishing the asymmetry of the V_memb_ amplitude in the high-signal subthreshold domain (Fig. 4*A*). By contrast, the CA1 neuron model (*model 3*), showed more phase lead and a higher synchrony point for depolarization compared with hyperpolarization due to a combination of *I*_h_ and *I*_M,_ with *I*_M_ producing its own perithreshold phenomenological inductance which only influenced high amplitude depolarizations (Fig. 3*A* and Fig. 6). Blocking *I*_h_ in the L5b models pyramidal cell models, or *I*_h_ and *I*_M_ in the CA1 pyramidal cell model, converted the neuronal response from a nonlinear resonator, which integrated large excitatory and inhibitory signals differentially, to an RC-like integrator, which treated them equally. Similar conversions between integrator and resonator have been shown to underlie observed differences in the intrinsic properties of CA1 pyramidal neurons between in vitro and in vivo-like conditions (75). Noradrenaline has been proposed as a neuromodulatory mechanism to switch L5b neurons from resonators to integrators through interactions with HCN channels as a means to separate motor planning and action (3, 76).

Phenomenological inductance serves to compensate for delays in V_memb_ changes caused by membrane capacitance (46, 77). Our results in models of L5b pyramidal neurons predict that this compensation will be augmented for large inhibitory inputs and diminished for large excitatory inputs compared with smaller amplitude inputs that remain in the linear regime. In our model of CA1 pyramidal neurons with two resonant currents (*I*_h_ and *I*_M)_, we predict the reverse. Vaidya et al. (77) observed increased phenomenological inductance with increasing distance from the soma along the apical dendrite in accordance with the increasing density of HCN channel expression. Thus, there was more inductive compensation of capacitive delays for stimuli distal from the soma compared with those proximal. Vaidya et al. (77) posited that the increasing phenomenological inductance with distance from the soma would ensure synchronous synaptic stimuli are simultaneously integrated at the soma, regardless of the synapse location – a “democracy of synapses” (77). Due to the differences in I/O phase shifts in response to excitation and inhibition described here, and the increased differences in phenomenological inductance with increased distance from the soma, our results suggest that integration of large amplitude stimuli will be undemocratic.

Although Φ^+^ changed smoothly with suprathreshold chirp stimulus during spike blockade in *model 1* (Fig. 2*C*), chirp stimulation was of limited utility for characterizing phase relationships with spiking (Fig. 2*D* and Fig. 8). This shortcoming was due to the combination of cell nonstationarity with the nonstationarity of chirp input. The I/O phase relationships for spiking identified by chirp predicted only the steady-state $\Phi_{n}^{+}$ (Fig. 8). For an 8 Hz stimulus, steady-state $\Phi_{n}^{+}$ is not achieved for nearly 1 s—a long time in terms of brain information processing.

We identified two patterns of spiking in response to continuing sinusoidal stimuli: *1*) “phase retreat,” in which spikes or bursts occurred progressively later in the cycles of the input stimulus (Fig. 7) and *2*) “phase advance,” in which spikes or bursts occurred progressively earlier in the cycles of the input stimulus (Fig. 9). Phase retreat emerged from spike-frequency adaptation – *I*_h_ and *I*_AHP_ were required to produce the gradual increase in spike threshold, driving phase shift $\Phi_{n}^{+}$ lower until reaching steady-state. Phase advance, on the other hand, emerged as the result of a sinusoidal stimulus whose amplitude increased over time, with the phase shift $\Phi_{n}^{+}$ increasing over time. Phase advance was also strongly modulated by, but not dependent on, *I*_h_ and *I*_AHP._ Both phenomena were also affected by stimulus location, frequency, and amplitude. Phase advance and phase retreat represent modes of spiking activity in which rate coding and temporal coding coexist. In both cases, information about the stimulus frequency is encoded in the spike rate. Information about the stimulus duration is encoded by $\Phi_{n}^{+}$ during phase retreat, and information about the stimulus amplitude, whether due to synaptic potentiation, presynaptic population recruitment, or increased presynaptic firing rate, is encoded by $\Phi_{n}^{+}$ during phase advance.

Phase shift relative to the local field potential (LFP) provides a mechanism for temporal/phase coding to accompany and complement rate coding in the central nervous system (CNS) (11–13, 78–80). Responses to optogenetic stimulation depend on the phase of stimulus activation (81–83); phase reset is an important aspect of phase determination and network behavior that will be affected by nonstationarity at the cellular scale (84–87). CNS phase coding has been most thoroughly studied in the context of “phase precession” in hippocampal place cells but has also been seen in entorhinal grid cells (53), as well as in successive theta waves in medial prefrontal cortex (88), and ventral striatum (89). In hippocampal place cells, the firing rate provides a coarse-grained encoding of the animal’s location in an arena (21). During phase precession, spiking occurs earlier on successive cycles of the theta wave as an animal moves towards the center of a place field, providing a finer-grained temporal encoding of location within the place field (54). Large pyramidal neurons in all of these areas have much in common in their impedance profiles, expression of HCN channels, and morphological features (23–25, 90). By contrast, a recent study identified “phase roll” in hippocampal CA1 neurons, with phase moving in the other direction to later times relative to theta (55).

While phase precession has frequently been modeled as an emergent property of the network (48–52), we suggest here that intrinsic cell properties would also play a role, enhancing the network effects both in hippocampus and cortex (91–93). In this view, phase advance would increase phase precession with increased sensory input, while phase retreat would produce phase roll in other cells, with steady inputs during the same theta period. The notion that movement through the place field results in increased strength of sensory-related inputs to pyramidal/place cells is common in models of phase precession (13, 78, 94). Network action would augment intrinsic effects by providing oscillatory excitatory stimulus to dendrites with inhibitory stimulus in the soma (13, 78, 80, 94, 95). Our results predict that phase precession could be tuned through modulation of *I_h_*, *I*_M_, or *I*_AHP_ (Fig. 9*B*, Supplemental Fig. S7*B*). Our model predicts that intrinsic aspects of both phase precession and phase roll could be tested by intracellularly applied blockers (intracellular to avoid network effects): precession would be enhanced by blocking either *I*_h_ or *I*_AHP;_ roll would be eliminated by either.

The modulation of subthreshold and suprathreshold I/O phase relationships in theta-resonant pyramidal neurons by a number of ion channel conductances makes them susceptible to channelopathies. Alterations in *I*_h_ in hippocampal pyramidal cells are associated with multiple forms of epilepsy (96–98); alterations in *I*_h_ and *I*_A_ in pyramidal cells are associated with Fragile X syndrome (99, 100); a number of genetic mutations known to alter *I*_M_ are associated with benign familial neonatal convulsions, epilepsy, and cognitive deficits (101); and genetic mutations increasing SK channel sensitivity to Ca^2+^ result in Zimmerman–Laband syndrome, which is associated with cognitive delay and developmental disability (102). Each of these pathological alterations will affect neuronal I/O phase relationships, which may contribute to the cognitive deficits associated with them.

The hypothesis of coexisting causes for phase precession comes as no surprise in the complex multiscale dynamical systems of the brain (103, 104). The two hypotheses – network cause and single-cell cause – are complementary. Hence, there is no falsifiability through eliminating one from the system as there would be with a physics hypothesis. Furthermore, eliminating intrinsic effects on a large scale (not just a single cell) or eliminating network effects will produce a new dynamical system that will not speak to either hypothesis: e.g., eliminating the network through block of all synaptic connections (locally, so as not to kill the animal), will eliminate precession trivially. The difficulties of multiscale modeling, and multiscale concepts, in biology are seen to be of a different order than those in many fields because of the failure of scale-by-scale encapsulation (105, 106). Famously, the individual atoms of a gas can be encapsulated as particles for the purposes of understanding the gas laws and thermodynamics: there is no need to consider electron shells or intranuclear forces. By contrast, the scale of a particular ion channel type (e.g., *I*_h_) cannot be fully encapsulated as a phenomenological inductor for the purpose of understanding neuronal membrane dynamics; the scale of the single neuron cannot be fully encapsulated as a sum-and-squash point neuron for understanding network dynamics; the scale of thalamus cannot be encapsulated as part of thalamocortical dynamics; *etc*. Failure of encapsulation, a complication of scale overlap, is seen throughout biology and manifests particularly clearly in the brain. Identifying interactions and emergences across spatiotemporal scales is the key to understanding the dynamics and behaviors of neural systems.

## DATA AVAILABILITY

The model is publicly available at https://github.com/suny-downstate-medical-center/nonlinear_impedance_phase.

## SUPPLEMENTAL DATA

10.6084/m9.figshare.22666648.v3 Supplemental Fig. S1: https://doi.org/10.6084/m9.figshare.22666648.v3;

10.6084/m9.figshare.23994303.v2 Supplemental Fig. S2: https://doi.org/10.6084/m9.figshare.23994303.v2;

10.6084/m9.figshare.23994321.v4 Supplemental Fig. S3: https://doi.org/10.6084/m9.figshare.23994321.v4;

10.6084/m9.figshare.23994336.v1 Supplemental Fig. S4: https://doi.org/10.6084/m9.figshare.23994336.v1;

10.6084/m9.figshare.23994345.v1 Supplemental Fig. S5: https://doi.org/10.6084/m9.figshare.23994345.v1;

10.6084/m9.figshare.23994351.v1 Supplemental Fig. S6: https://doi.org/10.6084/m9.figshare.23994351.v1;

10.6084/m9.figshare.23994357.v1 Supplemental Fig. S7: https://doi.org/10.6084/m9.figshare.23994357.v1.

## GRANTS

This research was funded in part by Aligning Science Across Parkinson’s [ASAP-020572; WWL] through the Michael J. Fox Foundation for Parkinson’s Research (MJFF).

## DISCLOSURES

No conflicts of interest, financial or otherwise, are declared by the authors.

## AUTHOR CONTRIBUTIONS

C.K., S.D.A., N.T.C., J.L.K., and W.W.L. conceived and designed research; C.K. performed experiments; C.K. analyzed data; C.K., S.D.A., N.T.C., J.L.K., and W.W.L. interpreted results of experiments; C.K. prepared figures; C.K. drafted manuscript; C.K., S.D.A., N.T.C., J.L.K., and W.W.L. edited and revised manuscript; C.K., S.D.A., N.T.C., J.L.K., and W.W.L. approved final version of manuscript.
